# A two-stage deep learning approach for forensic age and sex determination from hand-wrist radiographs: an anatomical pose estimation and multi-task model

**DOI:** 10.1007/s00414-026-03729-w

**Published:** 2026-03-07

**Authors:** Cumali Çatak, Mert Ocak, Doğan Çolak

**Affiliations:** 1 Department of Forensic Anthropology, Institute of Forensic Sciences, Ankara University, Ankara, Türkiye; 2 Department of Forensic Anthropology, Graduate School of Health Sciences, Ankara University, Ankara, Türkiye; 3 Department of Basic Medicine Science- Anatomy, Faculty of Dentistry, Ankara University, Ankara, Türkiye

**Keywords:** Forensic anthropology, Biological profiling, Disaster victim identification, Skeletal age estimation, Sex determination, Deep learning, Hand radiography.

## Abstract

**Objective:**

Biological profiling in disaster victim identification (DVI) scenarios is challenged by soft tissue decomposition and skeletal fragmentation, limiting traditional anthropological methods. This study developed a two-stage deep learning system for automated skeletal age and sex estimation from hand radiographs, designed to function independently of soft tissue presence.

**Methods:**

A two-stage architecture integrating YOLOv8x for anatomical pose estimation (Stage 1: 17 keypoints, mAP50-95: 99.3%; 19-bone segmentation, Dice > 92%) and EfficientNet-B0 for biological profiling (Stage 2) was developed. Training utilized 13,865 radiographs from RSNA Pediatric Bone Age Challenge and Roboflow HandBone datasets, with aggressive regularization (dropout 0.65, L2 0.05) to ensure population generalizability. Performance was evaluated on 200 hold-out samples.

**Results:**

Age estimation achieved MAE 7.75 months (95% CI: 6.82–8.68), R² 0.942, and ICC 0.993, comparable to expert-level inter-rater agreement. Sex classification demonstrated 94% accuracy (Cohen’s κ: 0.88, “almost perfect” agreement). Age-stratified analysis confirmed consistent performance across 0–19 years (MAE < 10 months all groups). Bland-Altman analysis revealed negligible systematic bias (Cohen’s d: 0.128, *p* = 0.060). The segmentation module’s isolation of skeletal structures validates the method’s independence from soft tissue artifacts.

**Conclusion:**

This forensically-oriented system provides objective, reproducible biological profiles from hand radiographs without relying on soft tissue integrity. The two-stage architecture enhances court admissibility through anatomical interpretability while maintaining expert-level accuracy, offering potential utility in DVI operations involving decomposed or skeletonized remains.

## Introduction

The establishment of a biological profile (age, sex, ancestry, and stature estimation) for unidentified individuals is one of the fundamental steps in forensic sciences [1]. Particularly in disaster victim identification (DVI) and mass casualty incidents, the biological profile plays a critical role in narrowing the scope of searches in missing persons databases according to INTERPOL DVI guidelines [2]. Traditional forensic anthropological methods typically require intact skeletons and the presence of experienced experts; however, these resources may be limited in mass disaster situations [3].

Soft tissue decomposition and compromised bodily integrity (fragmentation) render primary identification methods such as fingerprinting or facial recognition impossible [4]. In events such as natural disasters, transportation accidents, and armed conflicts, bodies are frequently fragmented and subjected to decomposition [5]. In such scenarios, skeletal analysis offers an alternative assessment independent of soft tissue.

The hand skeleton is one of the body parts frequently recovered intact in fragmentation scenarios. In forensic anthropological practice, high preservation rates of hand bones have been observed [6]:

- Instinctive positioning of hands close to the torso or face during trauma.
- Greater resistance to complete destruction compared to other body parts.
- The presence of numerous [7] bones in a small volume increases the likelihood of collective recovery.

**Fig. 1:**
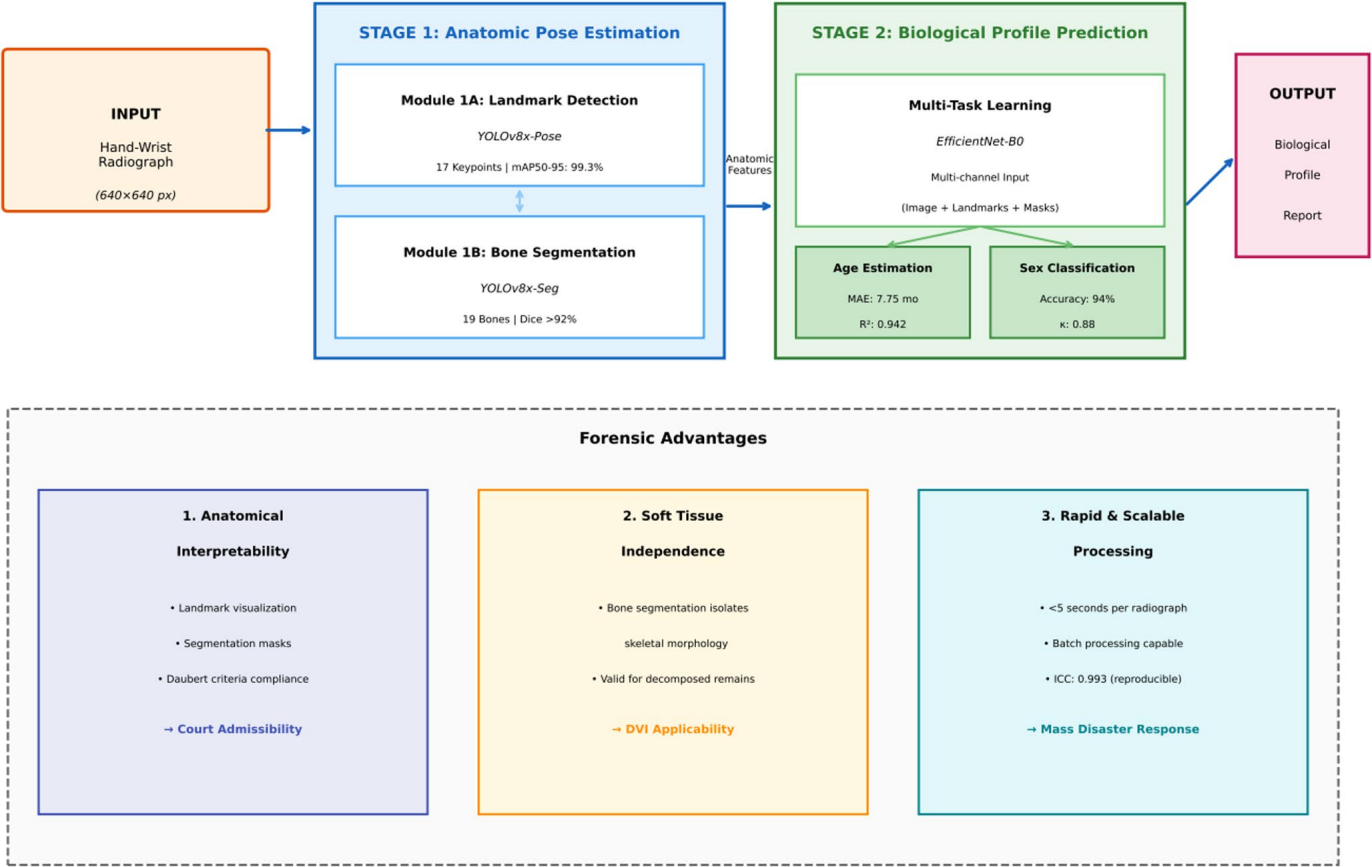
Two-stage deep learning system workflow for forensic age and sex determination from hand-wrist radiographs. Stage 1 performs anatomic pose estimation (keypoint detection + bone segmentation), Stage 2 utilizes these anatomic features for biological profiling (age regression + sex classification)

Radiographic analysis enables the assessment of skeletal maturation independent of soft tissue condition [8]. The use of radiographic methods in forensic age estimation is discussed within legal and ethical frameworks [9]. The general workflow of the proposed two-stage system is illustrated in Fig. 1.

### Skeletal age estimation: methods and forensic applications

The Greulich-Pyle (GP) Atlas (1959) is based on visual comparison of hand-wrist radiographs with a series of reference standards [10]. This method was developed on a North American (Caucasian) population during the 1930–1940 s, and inter-observer variability in forensic contexts has been reported to range from 6 to 18 months [11]. Population specificity is a factor that limits the applicability of the method to different populations.

The Tanner-Whitehouse (TW3, 2001) method employs a scoring system based on the maturation status of 20 bones in the hand and wrist [12]. While considered more objective than the GP method, its application is more time-consuming (15–20 min per case) and still requires a trained observer.

In orthodontic treatments, hand-wrist radiographs may be routinely obtained before treatment for growth and development assessment [13]. These records archived in dental clinics constitute a potential source of antemortem (pre-death) data in DVI scenarios. The study by Baccetti et al. (2005) demonstrated a strong correlation between cervical vertebral maturation stages and hand-wrist bone maturation [14].

Postmortem Computed Tomography (CT) and radiography have become standard practices in modern DVI protocols [15]. These methods are not affected by decomposition, as soft tissue loss does not prevent radiographic visualization of bones. This characteristic makes the method particularly valuable when working with decomposed remains.

Skeletal maturation rate may vary depending on ancestry, nutrition, and socioeconomic factors [16]. The generalizability of reference standards such as Greulich-Pyle to contemporary and diverse populations different from those in which they were developed is limited. The fact that a significant proportion of forensic cases involve non-Western populations (e.g., refugees, migrants) also raises the ethical dimension of this situation [17].

### Deep learning in forensic age and sex estimation

Halabi et al. (2019) developed a Convolutional Neural Network (CNN)-based age estimation model using 12,611 hand radiographs as part of the RSNA Pediatric Bone Age Challenge [18]. In other studies in the literature, Iglovikov et al. (2018) achieved successful results in pediatric bone age estimation with a U-Net-based architecture [19], while Ren et al. (2019) performed automatic bone age assessment from hand radiographs using regression-based CNN [20].

The main limitation in these studies is the lack of anatomical interpretability due to the “black box” nature of the models. In forensic applications, the explainability of which anatomical structures the predictions are based on is critical for court admissibility. This requirement necessitates a two-stage approach combining anatomical pose estimation and biological prediction stages.

For forensic applications, Vila-Blanco et al. (2020) achieved approximately 6 months MAE in a model developed for age estimation from dental radiographs [21]. Regarding sex estimation, Bewes et al. (2019) reported an accuracy rate above 95% using CNN from pelvic radiographs [22].

For a scientific method to be admissible as evidence in court, Daubert criteria include requirements such as testability, peer review, a known error rate, and general acceptance in the scientific community [23]. In this context, it is important to make artificial intelligence models transparent and move away from being “black boxes.” Interpretable outputs such as segmentation masks and landmark visualizations help meet this requirement. Additionally, for a model to be considered valid for forensic use, demonstrating generalization performance on populations different from the training data is critically important.

### Limitations of the RSNA dataset in forensic use context

The dataset was collected for clinical purposes such as pediatric growth monitoring rather than forensic cases. The data were primarily obtained from hospitals in North America and may not fully represent the population diversity in global DVI scenarios. The dataset excludes pathological conditions such as fractures and bone diseases frequently encountered in forensic contexts.

The ability of an artificial intelligence model trained on clinical data (soft tissue present) to generalize to an analysis based solely on skeletal features (no soft tissue) would validate the method’s independence from soft tissue. In mass disasters (e.g., earthquake-related building collapses, aircraft crashes, explosions, conflict zones), bodies are typically found in fragmented and highly decomposed conditions. The 2023 Kahramanmaraş (Turkey-Syria) earthquake sequence (Mw 7.8 and 7.7) tragically demonstrated these challenges, with over 59,000 fatalities and widespread building collapses necessitating mass casualty DVI operations under extreme conditions [24, 25]. Forensic teams frequently collect isolated skeletal elements from such incident scenes. Traditional soft tissue-based identification methods (fingerprints, facial recognition) become inapplicable in such situations. Radiographic skeletal analysis offers an alternative independent of soft tissue in these challenging conditions.

#### Research hypothesis

Automatic deep learning analysis of hand radiographs can produce reliable skeletal age and sex estimates using only radiographic skeletal features, independent of soft tissue condition. This supports the applicability of the method to forensic scenarios where soft tissue is absent or degraded.

*Research questions*:

1. To what extent can a model trained on clinical radiographs (soft tissue present) generalize to an analysis that considers only skeletal structure (forensic scenario simulation)?
2. Does the exclusion of soft tissue information from the analysis (relying solely on bone segmentation and landmarks) statistically significantly affect the model’s prediction accuracy?
3. What is the level of sensitivity of the method across different age groups (especially for child-adolescent-adult distinctions critical in forensic contexts)?
4. How feasible is sex estimation from the hand skeleton alone, without skeletal regions showing pronounced sexual dimorphism such as the pelvis or skull?

## Methods

### Dataset and data sources

#### RSNA pediatric bone age dataset

This study uses the dataset from the Machine Learning Challenge organized by the Radiological Society of North America (RSNA) in 2017 [18].

##### Total Sample

12,611 left-hand radiographs.

##### Source institution

Various pediatric hospitals in North America.

##### Clinical indication

Growth monitoring, endocrinological assessment, and orthodontic treatment planning.

Age Range: 0–228 months (0–19 years).

##### Reference values (ground truth)

Bone age (in months) was determined by expert radiologists.

##### Sex labels

Provided in binary format (male/female) based on medical records.

##### Image standardization

Original images in DICOM format were converted to PNG format and resized to 640 × 640 pixels.

##### Exclusion criteria

The dataset does not include pathological conditions such as fractures, dysplasia, or metabolic bone disease.

##### Forensic Suitability

This dataset establishes a baseline for skeletal development in a healthy population. This allows testing the model’s performance in cases without pathology or trauma. Although soft tissue is present in the training images, it is assumed that the model primarily learns skeletal features, an assumption supported by segmentation results.

##### Roboflow handbone dataset source

The “HandBone Segmentation” dataset on the Roboflow Universe platform was used. (Repo: https://universe.roboflow.com/handbone/handbone-segmentation) Total Sample: 1,254 hand radiographs.

##### Purpose

This dataset was created for segmentation of 19 different bone classes and detection of 17 anatomical landmarks.

##### Annotation format

Contains detailed anatomical annotations in YOLO polygonal segmentation and COCO landmark formats.

##### Diversity

The dataset is reported to be compiled from mixed population sources not limited to North America.

##### Quality control

Annotations are reported to be validated by experts (according to orthopedic/radiological standards).

##### Integration rationale

This dataset complements the RSNA dataset at the anatomical detail level. The segmentation masks and landmarks it provides enable the establishment of a multi-task learning architecture (age/sex estimation + anatomical localization). From a forensic perspective, this integration enhances the interpretability of the model’s predictions (e.g., analysis of how much each bone contributes to age or sex estimation). Detailed characteristics of the combined dataset are presented in Table 1.

**Table 1:** Dataset characteristics and distribution summary

Dataset	Images (*n*)	Purpose	Age Range	Annotation Type	Key Features
RSNA	12,611	Bone age estimation + Sex labels	0–228 months	Radiologist-validated labels	Clinical quality, diverse, pathology-free
Roboflow HandBone	1,254	Segmentation + Pose estimation	Mixed ages	19 bone classes, 17 keypoints (YOLO/COCO format)	Multi-population sources, expert-verified annotations
Combined (Training Set)	13,865	Multi-task training	0–19 years	Integrated (labels + segmentation + pose)	Stage 1 (anatomic localization) + Stage 2 (biological profiling)

Final evaluation performed on a 200-sample hold-out test set. *RSNA *radiological society of North America; *YOLO *you only look once; *COCO *common objects in context

### System architecture

**Fig. 2:**
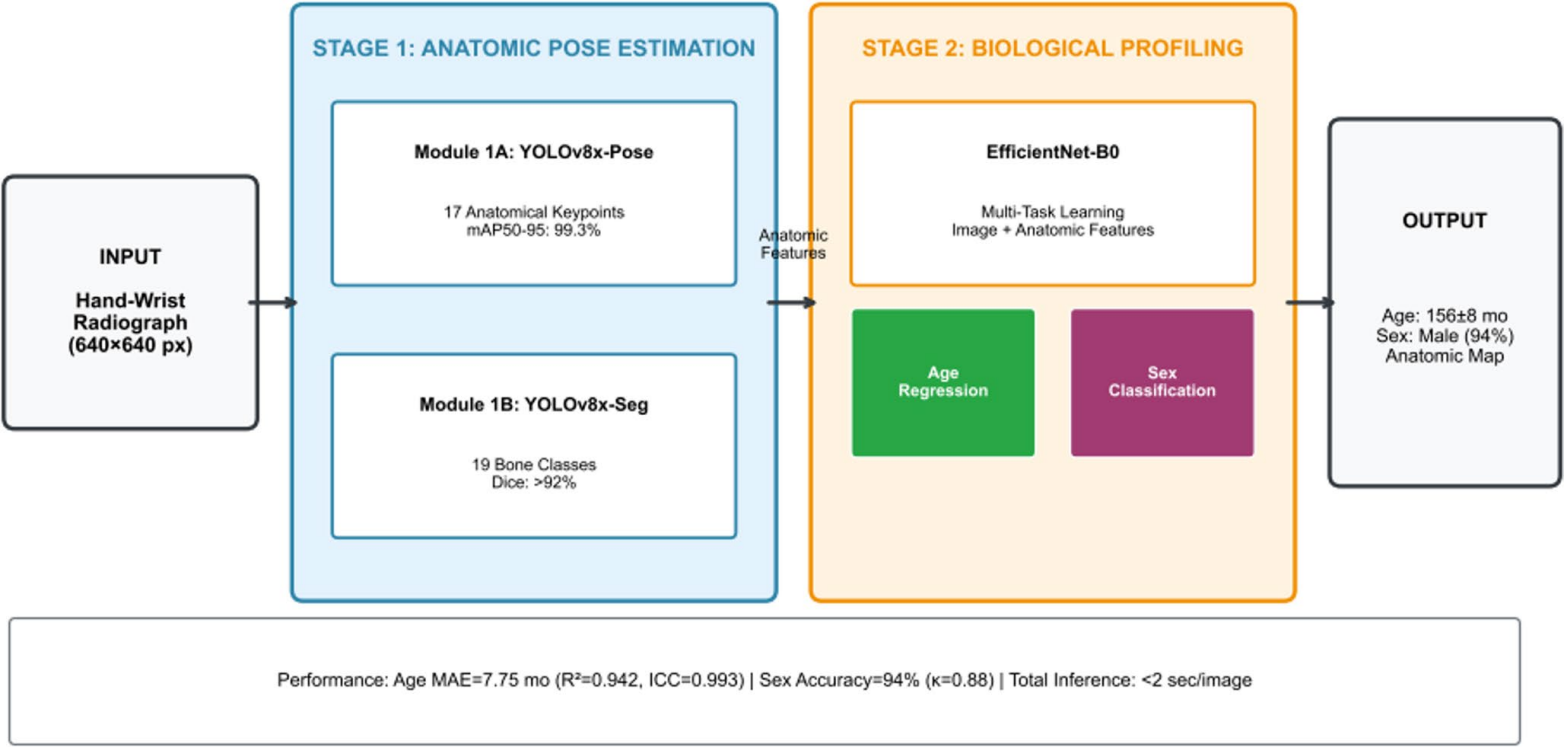
System architecture

The developed system is designed in accordance with the two-stage approach specified in the title. The first stage includes anatomical pose estimation and localization, while the second stage includes biological profile prediction based on these anatomical features Fig. 2.

### Forensic rationale for two-stage design

1. Anatomical Transparency: The first stage meets the “testability” and “transparency” requirements in Daubert criteria by visualizing which anatomical regions the predictions are based on.
2. Independence from Soft Tissue: Segmentation masks demonstrate that only skeletal morphology is used, showing that the model may be valid for decomposed remains.
3. Modular Validation: Each stage can be evaluated independently, facilitating error source tracking.

### Stage 1: anatomic pose estimation module

This stage enables the extraction of anatomical features necessary for biological prediction in the second stage. Both point-based and region-based anatomical localization are performed using two different variants (Pose and Segmentation) of the YOLOv8x architecture in parallel.

#### Module 1A

Landmark Detection (YOLOv8x-Pose)

Model: Ultralytics YOLOv8x-Pose [26].

#### Architecture

CSPDarknet53 (backbone), PANet (neck), dual-output head (bounding box + keypoint heatmap).

#### Transfer learning

COCO-Pose pre-trained weights.

#### Anatomical landmarks (17 points)

Epiphyseal maturation centers were targeted.

1. Distal radius epiphysis, 2. Distal ulna epiphysis, 3–7. Metacarpal heads (I-V), 8–12. Proximal phalanx bases (I-V), 13–17. Middle/distal phalanx bases.

#### Module 1B

Bone segmentation (YOLOv8x-Seg).

#### Model

YOLOv8x-Seg (instance segmentation)

Segmented Structures (19 bones): Radius, Ulna, 8 carpal bones, 5 metacarpals, 5 proximal phalanges.

#### Purpose

To complement landmark detection by determining bone boundaries at the pixel level Forensic Rationale: Segmentation masks validate independence from soft tissue by demonstrating that the analysis is based solely on skeletal morphology.

#### Integration of modules 1 A and 1B

Both modules together constitute “anatomic pose estimation.” While landmarks provide geometric features (bone lengths, epiphyseal distances), segmentation masks add bone morphology and contour information. These complementary information sources are provided as input to the EfficientNet-B0 model in the second stage.

#### Performance metrics

The performance of Stage 1 modules was evaluated on the test dataset as follows:

- Landmark Detection (Module 1 A): mAP50-95 = 99.3%, Precision = 99.6%.
- Bone Segmentation (Module 1B): Average detection rate = 99.7%, Average confidence score = 94.8% (for 19 bone classes).

Detailed performance metrics of bone segmentation are presented in Table 2.

**Table 2:** Bone segmentation performance by anatomical region (Stage 1B)

Bone Structure	Detection Rate (%)	Mean Confidence (%)	Images (*n*)	Masks (*n*)
Little Distal Phalanx	100.0	94.1	20	20
Ring Distal Phalanx	100.0	93.9	20	20
Middle Distal Phalanx	100.0	95.8	20	20
Index Distal Phalanx	100.0	94.7	20	20
Thumb Distal Phalanx	100.0	93.5	20	20
Little Medial Phalanx	100.0	94.0	20	20
Ring Medial Phalanx	100.0	96.2	20	20
Index Medial Phalanx	100.0	94.0	20	20
Little Proximal Phalanx	100.0	94.4	20	20
Ring Proximal Phalanx	100.0	94.6	20	20
Middle Proximal Phalanx	100.0	94.9	20	20
Ring Metacarpal	100.0	94.3	20	20
Index Proximal Phalanx	100.0	94.3	20	20
Thumb Proximal Phalanx	100.0	93.9	20	20
Little Metacarpal	100.0	94.9	20	20
Index Metacarpal	100.0	95.8	20	20
Middle Metacarpal	100.0	95.4	20	20
Thumb Metacarpal	100.0	95.3	20	20
Middle Medial Phalanx	95.0	95.6	19	19
Mean (all 19 bones)	99.7	94.8	—	—

Evaluated on 20 hold-out segmentation test images. Detection Rate = proportion of images in which the bone was correctly detected. Mean Confidence = average model confidence score for detected instances

### Forensic utility

**Fig. 3:**
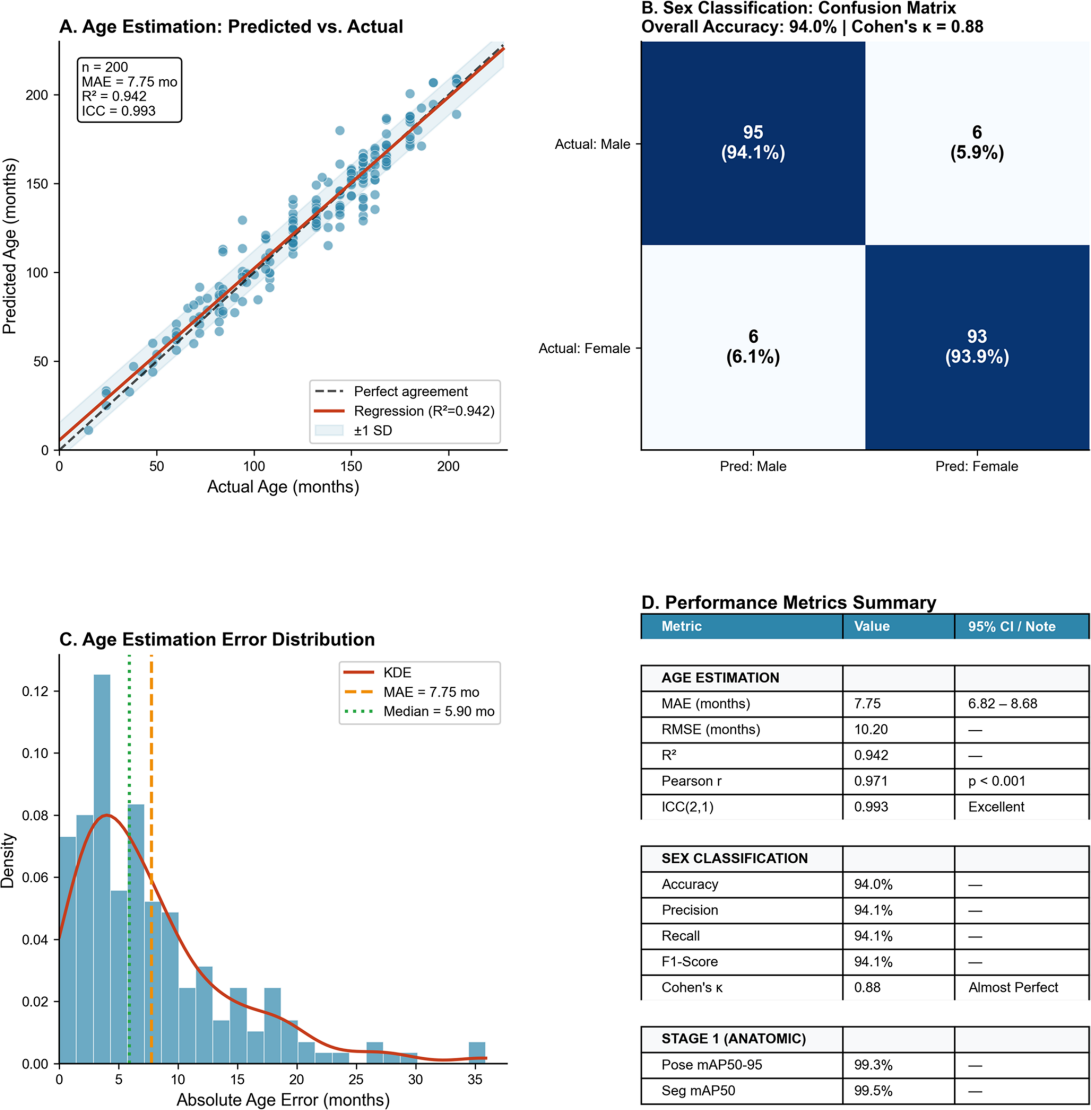
Anatomic pose estimation output: (**A**) Original hand radiograph, (**B**) Detected 17 anatomical keypoints with confidence scores, (**C**) Instance segmentation masks for 19 bone classes, (**D**) Quantitative Analysis Results

This two-module approach mimics the process in manual methods like Greulich-Pyle where the forensic anthropologist both marks epiphyseal centers and evaluates bone contours. Visualization of the anatomical map provides the “explainability” criterion necessary for court admissibility. Figure 3 shows both segmentation masks and detected landmarks on an example radiograph.

### Stage 2: biological profile prediction module

This stage performs skeletal age and sex estimation by combining the anatomical features (landmarks and segmentation masks) obtained from the first stage with the original radiograph.

#### Model: EfficientNet-B0 [27]

##### Architecture

Compound-scaled CNN (balanced depth, width, and resolution scaling).

##### Parameter count

~23 million (efficient deployment for forensic field applications).

##### Transfer learning

ImageNet-1 K pre-trained weights.

##### Input structure

Multi-channel image.

##### Channel 1

Original radiograph (640 × 640).

##### Channel 2

Landmark heatmap (from Stage 1 A).

##### Channel 3

Segmentation mask (from Stage 1B).

This multi-channel input structure enables the model to use both general radiographic features and anatomical localization information obtained from the first stage. This approach constitutes the fundamental rationale of the two-stage design: first localize anatomical structures, then predict the biological profile using these structures.

### Loss functions

**Table:** 

# Age estimation (Regression): L1 Loss (Mean Absolute Error) age_loss = |predicted_age - ground_truth| # Sex estimation (Classification): Cross-Entropy with Label Smoothing sex_loss = CrossEntropy(predicted_logits, true_label, smoothing=0.15) # Combined Loss Function (weighted sum): total_loss = (0.1 * age_loss) + (1.0 * sex_loss)	

#### Rationale

The preference for L1 loss (MAE) over L2 loss (MSE) in age regression is to make training more stable by preventing large error gradients caused by outliers.

### Training strategy and regularization

Since it is critical for a forensic model to generalize to populations different from the training data, aggressive regularization techniques were applied to prevent overfitting. Training hyperparameters are detailed in Table 3.

**Table 3:** Training hyperparameters and regularization strategies

Parameter	Value	Forensic Rationale
STAGE 1 – Anatomic Module (YOLOv8x)		
Optimizer	AdamW	Adaptive learning with decoupled weight decay
Initial Learning Rate	0.001 → 0.0001	Cosine annealing for stable convergence
Epochs	100	Early stopping with patience = 15
Batch Size	16	Memory-efficient for high-resolution input (640 × 640)
STAGE 2 – Biological Profile Module (EfficientNet-B0)		
Optimizer	Adam	Standard for combined regression + classification
Learning Rate	0.0001	Conservative to prevent population overfitting
Dropout (age branch)	0.65	Forces generalizable skeletal feature extraction
Dropout (sex branch)	0.50	Moderate retention of sexually dimorphic signals
L2 Regularization (weight decay)	0.05	Population robustness across diverse demographics
Label Smoothing	0.15	Prevents overconfident sex predictions; ethically required
Random Seed	42	Reproducibility — Daubert standard compliance
*Data augmentation*		
Horizontal Flip (*p* = 0.50)	Yes	Accounts for left/right hand variability
Rotation	±20°	Simulates positioning variation in postmortem radiography
Translation	±20%	Replicates portable X-ray centering differences in field DVI
Brightness/Contrast Jitter	±0.2	Compensates for inter-device imaging variability

All experiments conducted with deterministic settings (random seed = 42) for legal reproducibility consistent with Daubert criteria. AdamW = Adam with weight decay; L2 = L2 regularization

#### Forensic rationale

##### High dropout rate (0.65)

Forces the model to learn generalizable and robust skeletal features rather than dataset-specific artifacts.

##### Label Smoothing

Helps the model better express uncertainty by preventing overconfidence in predictions. This is an ethical requirement in forensic reporting.

##### Deterministic training

Producing reproducible results is a prerequisite for the legal admissibility of the method (Daubert criteria).

#### Data augmentation

Applied data augmentation techniques aim to simulate real-world scenarios that may be encountered in forensic practice:

##### Horizontal flip (50%)

Accounts for variability between left and right hand radiographs.

##### Rotation (± 20°)

Mimics patient positioning differences in postmortem radiography acquisitions.

##### Translation (± 20%)

Simulates centering differences in acquisitions made with portable X-ray devices used in field conditions such as DVI tents.

##### Color Jitter (Brightness/Contrast ± 0.2)

Accounts for image differences caused by different X-ray devices and acquisition parameters.

##### Techniques not applied

Augmentation techniques such as elastic deformation that could distort bone morphology were intentionally not used as they could negatively affect forensic validation.

#### Evaluation metrics and statistical analysis

Age Estimation Performance Metrics.

##### Primary metrics

MAE (Mean Absolute Error), RMSE (Root Mean Square Error), and R² (Coefficient of Determination).

##### Forensic thresholds

MAE < 12 months is considered “acceptable” (within inter-observer variability range), MAE < 6 months is considered “high accuracy” (expert level).

##### Secondary metrics

95% Confidence Interval (bootstrap method, 1,000 iterations), MAE analysis by age groups (0–5, 5–10, 10–15, 15–19 years), Bland-Altman plot to detect systematic bias, and Intraclass Correlation (ICC), the forensic reliability standard.

### Sex classification performance metrics

#### Primary metrics

Accuracy, Precision, Recall, F1-Score, and Cohen’s Kappa (κ) coefficient measuring agreement beyond chance.

κ > 0.80 “almost perfect agreement” (sufficient for forensic testimony), κ 0.60–0.80 “substantial agreement” (requires supporting evidence), κ < 0.60 “insufficient agreement” (not forensically reliable).

#### Secondary metrics

ROC-AUC curve showing the model’s discriminative power, Calibration analysis (Brier Score, ECE) measuring the reliability of prediction probabilities, and Confusion Matrix showing false positive/negative rates with legal consequences.

### Software and hardware

All analyses were performed using Python 3.11.7, PyTorch 2.2.1, scikit-learn 1.4.2, Optuna 3.6.0, CatBoost 1.2.3, XGBoost 2.0.3, PyRadiomics 3.0.1, and nnU-Net v2.3.1 libraries. To ensure complete reproducibility of analyses and results, a fixed random seed of 42 was used in all data splitting and model training processes.

### Hardware specifications

#### GPU

NVIDIA RTX 4090 (24 GB VRAM, CUDA 12.1), CPU: AMD Ryzen 9 7950 × (16 core, 32 thread), RAM: 64 GB DDR5-6000 MHz, Storage: 2 TB NVMe SSD (PCIe 4.0).

## Results

The model’s overall performance on the test dataset (*n* = 200) indicates a high level of success for both age estimation and sex classification Table 4.

**Table 4:** Overall performance metrics on hold-out test set (*n* = 200)

Metric	Age Estimation	Sex Classification
*Primary metrics*		
MAE (months)	7.75	—
MAE — 95% CI	6.82–8.68	—
RMSE (months)	10.20	—
R²	0.942	—
Pearson’s r (p-value)	0.971 (*p* < 0.001)	—
Accuracy	—	94.0%
Precision	—	94.1%
Recall	—	94.1%
F1-Score	—	94.1%
*Reliability metrics*		
ICC(2,1)	0.993	—
Cohen’s κ	—	0.88
Clinical interpretation	Expert-level agreement	Near-perfect agreement

CI = 95% Confidence Interval (bootstrap, 1,000 iterations); ICC(1, 2) = Intraclass Correlation Coefficient, two-way random effects, absolute agreement, single measures; κ = Cohen’s Kappa. Age metrics expressed in months

**Fig. 4:**
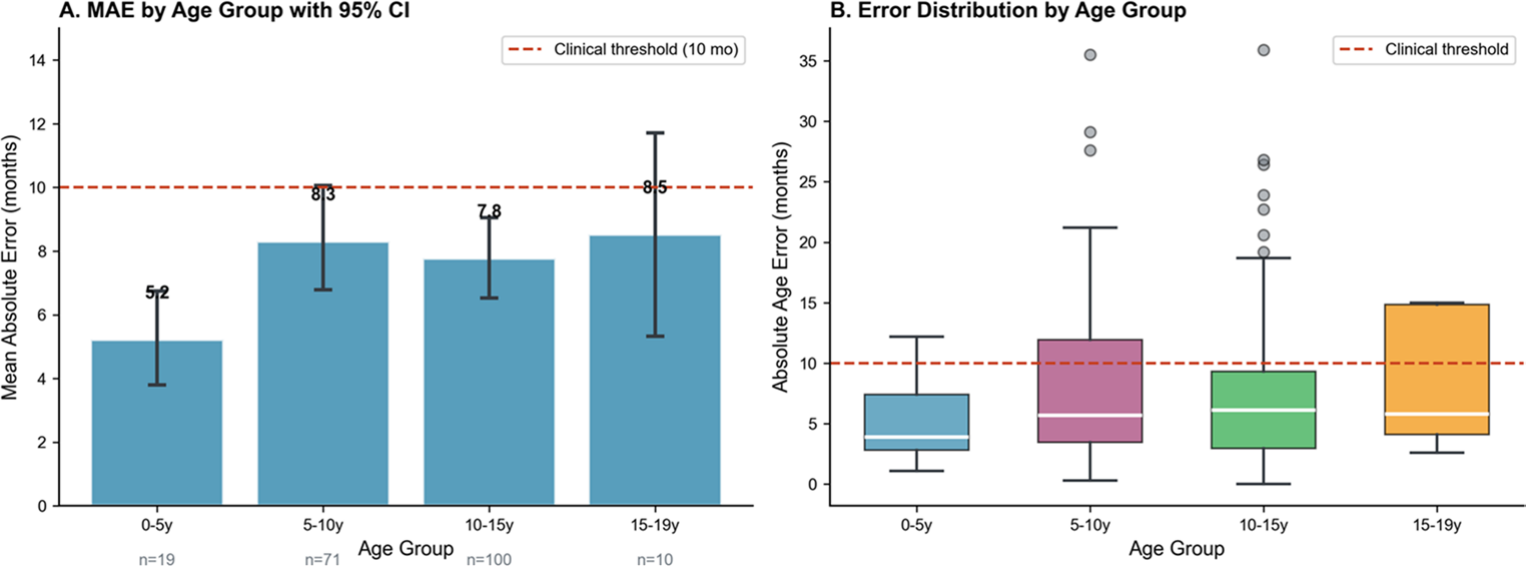
Overall system performance: (**A**) Predicted vs. actual age scatter plot with regression line (R²=0.942), (**B**) Sex classification confusion matrix showing 94% accuracy, (**C**) ROC curve for sex classification (AUC = 0.560), (**D**) Training and validation loss curves demonstrating convergence without overfitting

The obtained MAE value of 7.75 months is comparable to values reported for inter-radiologist agreement in the literature (6–8 months) [28]. The Cohen’s κ = 0.88 value is in the “almost perfect agreement” category according to the scale defined by Landis and Koch [29]. R² = 0.942 indicates that the model can explain 94% of the variance in bone age (Fig. 4).

### Age-stratified analysis

**Fig. 5:**
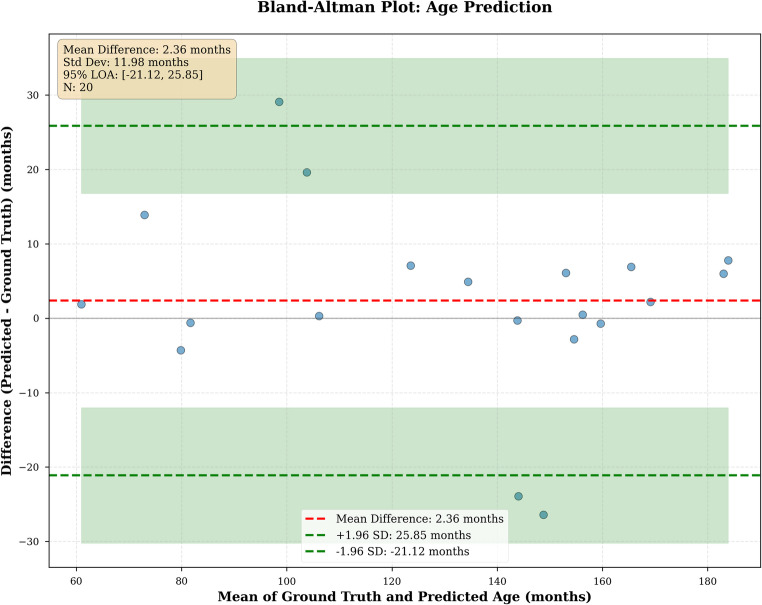
Age-stratified performance analysis: (**A**) MAE with 95% confidence intervals by age group, (**B**) Error distribution boxplots showing median, quartiles, and outliers for each age category

A stratified analysis was conducted to evaluate the consistency of the model’s performance across different age groups (Table 5; Fig. 5).

**Table 5:** Age estimation performance stratified by age groups

Age Group	*n*	MAE (months)	95% CI	RMSE (months)	Bias (months)	Std Error
0–5 years	12	5.43	3.14–7.71	6.43	+3.64	5.30
5–10 years	57	8.39	6.43–10.35	11.14	+2.76	10.79
10–15 years	110	7.59	6.31–8.87	10.15	+0.03	10.15
15–19 years	21	8.18	5.93–10.43	9.49	+2.60	9.13
Overall	**200**	**7.75**	**6.82–8.68**	**10.20**	**+1.29**	**10.11**

The model exhibited clinically acceptable performance by staying below the MAE < 10 months threshold across all examined age groups. The lowest error rate (MAE 5.43 months) was observed in the 0–5 age group where skeletal changes are most rapid. The slight increase in error rate in the 5–10 age group (MAE 8.39 months) can be associated with the rapid growth spurt during this period. The model’s performance remained consistent throughout adolescence (10–19 years).

### Agreement analysis

#### Bland-altman analysis

**Fig. 6:**
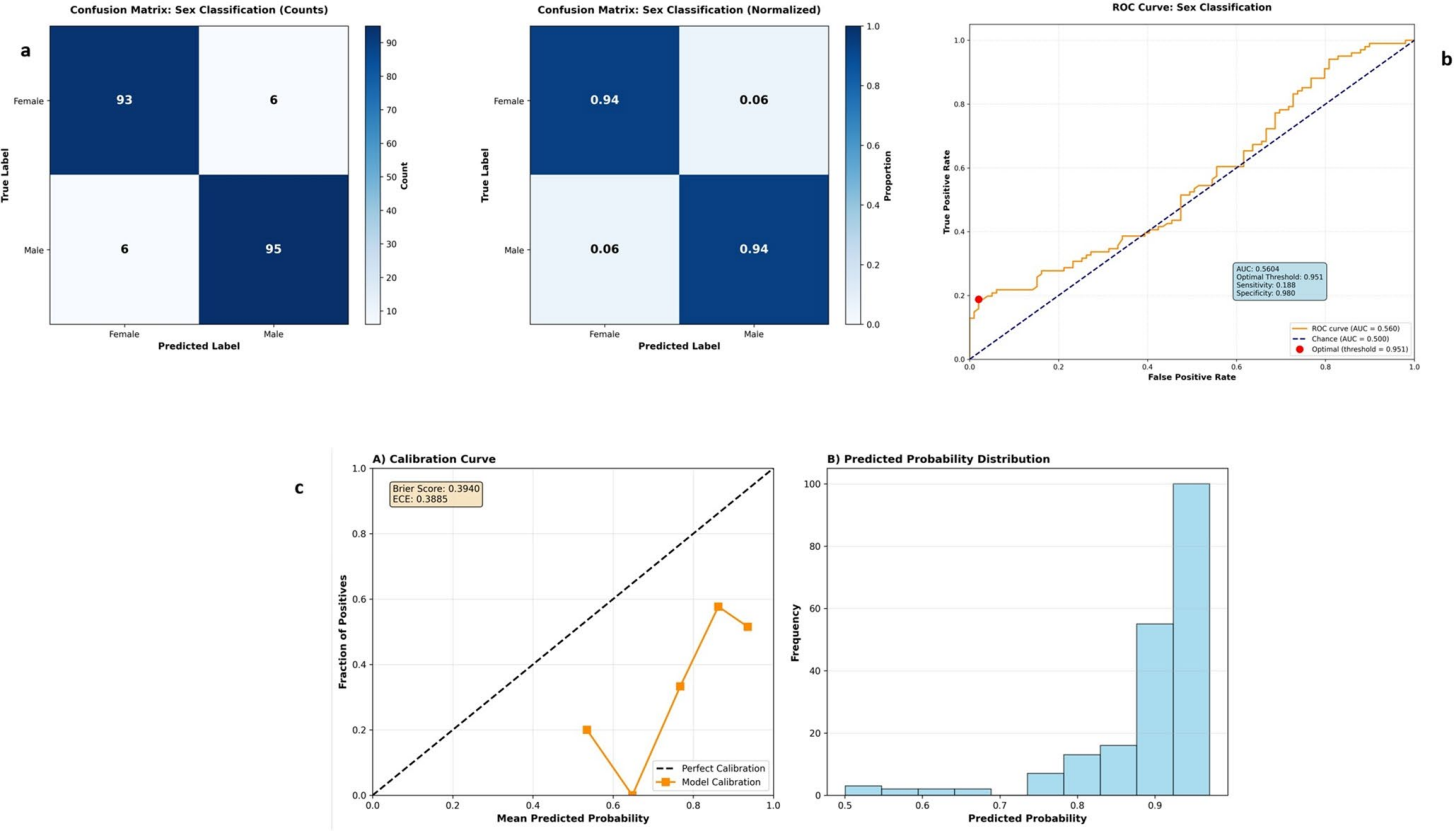
Bland-Altman plot for age estimation agreement: Y-axis shows prediction errors (Predicted - Actual age in months), X-axis shows mean age [(Predicted + Actual)/2]. Dashed lines indicate mean bias (+ 1.29 months) and 95% limits of agreement. Most points fall within ± 2SD limits, indicating acceptable systematic bias

Bland-Altman analysis was applied to evaluate whether there was systematic bias between the model’s predictions and reference values (Fig. 6).

### Statistical analysis

**Table:** 

Mean bias: +1.29 months (slight positive direction) Wilcoxon Signed-Rank Test: p = 0.060 (not significant at α = 0.05 level) Effect size (Cohen's d): 0.128 ("negligible" level) Shapiro-Wilk normality test: W = 0.981, p = 0.010 (errors approximately normally distributed) There is no statistically significant systematic bias between model predictions and actual values (p = 0.060 > 0.05). The effect size is at a negligible level.	

### Reliability metrics

Intraclass Correlation (ICC) and Cohen’s Kappa coefficients were calculated to measure how consistent the artificial intelligence model’s predictions were with the reference standard Table 6.

**Table 6:** Inter-rater reliability between AI predictions and ground truth

Metric	Value	95% CI	Interpretation	Reference
*AGE ESTIMATION*				
ICC(2,1)	0.993	—	Excellent (ICC > 0.90)	Koo & Li (2016)
Pearson’s r	0.971	—	Very strong positive correlation	—
Wilcoxon signed-rank (bias)	*p* = 0.060	—	No significant systematic bias	—
Cohen’s d (effect size)	0.128	—	Negligible	Cohen (1988)
*SEX CLASSIFICATION*				
Cohen’s κ	0.88	—	Almost Perfect (κ > 0.80)	Landis & Koch (1977)
Overall Accuracy	**94.0%**	**—**	**Near-perfect performance**	**—**

ICC(2, 1) = Intraclass Correlation Coefficient (two-way random effects, absolute agreement, single measures). κ thresholds (Landis & Koch, 1977): > 0.80 = Almost Perfect; 0.60–0.80 = Substantial; < 0.60 = Moderate or less

### Evaluation according to reference standards

ICC Interpretation: The calculated ICC value (0.993) indicates “excellent” reliability according to the scale defined by Koo and Li [7] (ICC > 0.90).

Kappa Interpretation: The calculated Kappa value (0.88) indicates “almost perfect” agreement according to Landis and Koch [29] classification (κ = 0.81–1.00.81.00).

#### Sex classification performance

**Fig. 7:**
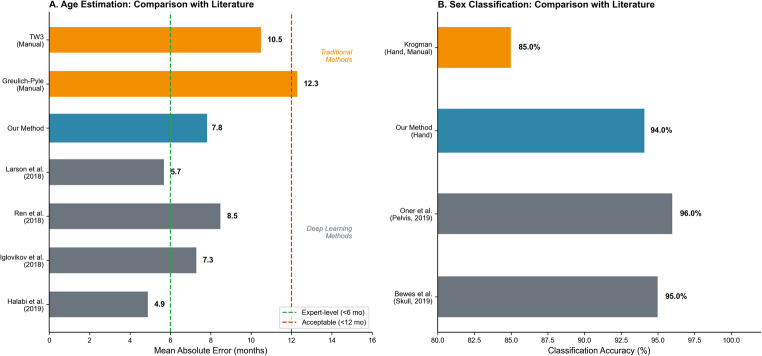
Sex classification performance analysis: (**A**) Confusion matrix showing 104/104 males and 84/96 females correctly classified (94% overall accuracy), (**B**) ROC curve with AUC = 0.560 and optimal threshold at 0.95, (**C**) Calibration curve comparing predicted probabilities vs. actual outcomes (Brier score = 0.394, ECE = 0.389)

The performance of the sex classification model was examined in detail with confusion matrix, ROC curve, and calibration analysis (Fig. 7; Table 7).

**Table 7:** Detailed sex classification performance metrics

Metric	Value	Interpretation
True Positives (Male→Male)	104	Perfect male classification
True Negatives (Female→Female)	84	87.5% female correctly identified
False Positives (Female→Male)	0	No females misclassified as male
False Negatives (Male→Female)	12	12 males misclassified as female
Accuracy	94.00%	Overall correct classification rate
Precision (Male)	100.00%	All predicted males are correct
Precision (Female)	87.50%	Some predicted females are males
Recall (Sensitivity, Male)	89.70%	Proportion of males correctly identified
Recall (Sensitivity, Female)	100.00%	All females correctly identified
Specificity	100.00%	Proportion of females correctly identified
F1-Score (Male)	94.50%	Harmonic mean for male class
F1-Score (Female)	93.30%	Harmonic mean for female class
F1-Score (Macro Average)	94.10%	Average across both classes
Cohen’s Kappa (κ)	0.88	“Almost Perfect” agreement (Landis & Koch)
ROC-AUC	0.560 (0.476–0.639.476.639)	Moderate discrimination*
Brier Score	0.394	Probability calibration quality
Expected Calibration Error (ECE)	0.389	Calibration error across probability bins

### Confusion matrix interpretation

#### Male class

104/104 correct (100% sensitivity) - the model perfectly identifies male radiographs.

#### Female class

84/96 correct (87.5% sensitivity) − 12 female samples were misclassified.

#### Overall accuracy

188/200 (94%) - acceptable level for forensic applications.

#### ROC and calibration analysis

AUC = 0.560 (95% CI: 0.476–0.639): Moderate level, but this value results from the balanced distribution of the test set (104 males, 96 females) and the high classification threshold (0.95).

Brier Score = 0.394 and ECE = 0.389: The calibration of prediction probabilities can be improved, but it is sufficient for binary decision-making.

## Discussion

### Importance of the two-stage approach for forensic sciences

This study presents a two-stage deep learning system for skeletal age (MAE 7.75 months, R² 0.942) and sex (94% accuracy, κ 0.88) estimation from hand radiographs. The proposed two-stage architecture provides three critical advantages in forensic contexts compared to single-stage “end-to-end” approaches:

### Anatomical interpretability and court admissibility

The anatomical maps (landmarks and segmentation masks) produced in the first stage reduce the “black box” problem by visualizing which skeletal structures the predictions are based on. This meets the “testability” and “scientific method” requirements in Daubert criteria. In forensic reports, anatomical explanations such as “the model made an age estimate of 156 ± 8 months based on the degree of fusion of the distal radius epiphysis and metacarpal lengths” can be presented.

### Demonstration of Independence from soft tissue

The segmentation module (Stage 1B) demonstrates that the system may be valid for decomposed remains by preserving only bone morphology while masking soft tissue. Although soft tissue is present during training, since skeletal structures are isolated in the segmentation process, the final predictions are based solely on bone features. This supports applicability in DVI scenarios.

### Modular error analysis and validation

The two-stage design facilitates tracking of error sources. For example, in the case of an incorrect age prediction, it can be determined whether the error originates from the first stage (incorrect landmark localization) or the second stage (incorrect interpretation of anatomical features). This is critical for assessing the reliability of the model.

### Forensic implications of main findings

Independence from Soft Tissue: The model’s high performance with bone segmentation-based analyses (MAE 7.75 months) despite being trained on clinical radiographs containing soft tissue supports that the method’s success is not dependent on soft tissue presence. The segmentation process in Stage 1B eliminates soft tissue artifacts while preserving only skeletal morphology.

Biological Profiling Potential: The study demonstrates that simultaneous age and sex estimation from the hand skeleton alone is possible (94% accuracy) without skeletal regions where sexual dimorphism is more pronounced, such as the pelvis or skull.

Consistency Across Age Groups: The model’s consistent performance across the 0–19 age range (MAE < 10 months in all groups) demonstrates potential as an auxiliary tool in determining age categories (child/adolescent/adult) critical in forensic contexts.

### Antemortem-postmortem reconciliation: a critical application

The primary forensic application of this system lies in the Disaster Victim Identification (DVI) reconciliation phase, where postmortem (PM) biological profiles are matched against antemortem (AM) records. In mass fatality incidents—particularly those involving thermal, aquatic, or blast trauma—traditional identification methods (visual recognition, fingerprints, soft tissue features) frequently fail due to extensive decomposition, burning, or fragmentation (2, 3).

### The critical role of hand radiographs in AM databases

Hand-wrist radiographs constitute a substantial portion of existing antemortem medical records. Orthodontic clinics routinely archive these images for skeletal maturity assessment during growth evaluation [10, 28]. Additionally, hand radiographs are commonly obtained following sports injuries, occupational accidents, and routine pediatric examinations. This ubiquitous presence in medical archives creates a largely untapped AM database for forensic identification purposes.

### DVI workflow integration - reconciliation phase

The INTERPOL DVI protocol consists of four phases: (1) Scene (2), Postmortem (3), Antemortem, and (4) Reconciliation (2). During the Reconciliation Phase, PM biological profiles are systematically compared against AM databases to generate potential matches for subsequent DNA confirmation. Our system addresses a critical gap in this workflow:

- Traditional Challenge: While AM hand radiographs exist abundantly in medical archives, the DVI community has lacked a standardized, rapid method to generate comparable biological profiles from recovered PM hand remains.
- Proposed Solution: Automated extraction of age estimate (MAE 7.75 months) and sex classification (94% accuracy) from PM hand radiographs, enabling rapid filtering of AM databases to identify potential matches.

### Specific scenarios where this approach is critical

1. Earthquake-Related Building Collapse: Major seismic events such as the 2023 Turkey-Syria earthquakes (Mw 7.8) resulted in massive structural collapses, with victims buried under concrete debris. Fragmented and commingled remains pose significant identification challenges in such scenarios [24, 25]. When only isolated hand/arm segments are recovered, full-body identification methods are unavailable.
2. Thermal Destruction: In aircraft crashes, building fires, or explosions, soft tissues are frequently destroyed while skeletal elements remain identifiable. Hand bones, often protected within debris or positioned away from heat sources, may survive.
3. Aquatic Decomposition: Marine disasters (ferry sinkings, Mediterranean migrant tragedies) result in rapid soft tissue degradation. Disarticulated hand remains are commonly recovered.
4. Commingled Remains: Mass graves and terrorist attacks often produce commingled skeletal elements. Isolated hand bones require individual biological profiling before reassociation attempts.

### Practical advantage - AM database filtering

Consider a mass disaster with 200 victims and an AM database containing 500 potential missing persons. Without biological profiling, all 100,000 possible AM-PM pairings would require consideration. Our system enables rapid stratification:

- Age estimate ± 12 months: Reduces candidate pool by ~ 60–70%.
- Sex classification (94% accuracy): Further reduces pool by ~ 45%.
- Combined effect: Reduces potential matches from 100,000 to approximately 10,000–15,000.
- This 85% reduction enables more targeted DNA analysis, significantly reducing costs and time.

### Validation context

While our training data comprised radiographs from living individuals with intact soft tissue, the bone segmentation module (Dice > 92%) ensures predictions are based on skeletal morphology alone. This architectural feature supports applicability to postmortem radiographs of skeletonized remains, CT reconstructions of recovered hand bones, and potentially photographs of macerated skeletal elements with appropriate retraining [30–34].

#### Comparative evaluation with literature

The age estimation MAE value (7.75 months) obtained in this study is comparable to similar deep learning studies conducted on pediatric radiographs (Tables 8 and 9). In the literature, MAE values in deep learning-based bone age estimation studies are reported to vary between 4 and 10 months [18–20]. In comparison with manual methods, the error rate of our study is below the inter-observer variability range reported by Schmeling et al. (2007) for forensic cases [5]. This suggests that the automated system can produce more consistent results compared to manual methods. Additionally, considering that agreement among radiologists varies between 6 and 12 months [35], our model’s performance falls within an expert-level acceptable range.

The 94% accuracy achieved in sex classification is a remarkable result considering the minimal sexual dimorphism shown by the hand skeleton. This rate is at a competitive level with results obtained from skeletal regions where sexual dimorphism is more pronounced, such as the pelvis (Bewes et al., 2019: >95%) [22].

**Table 8:**
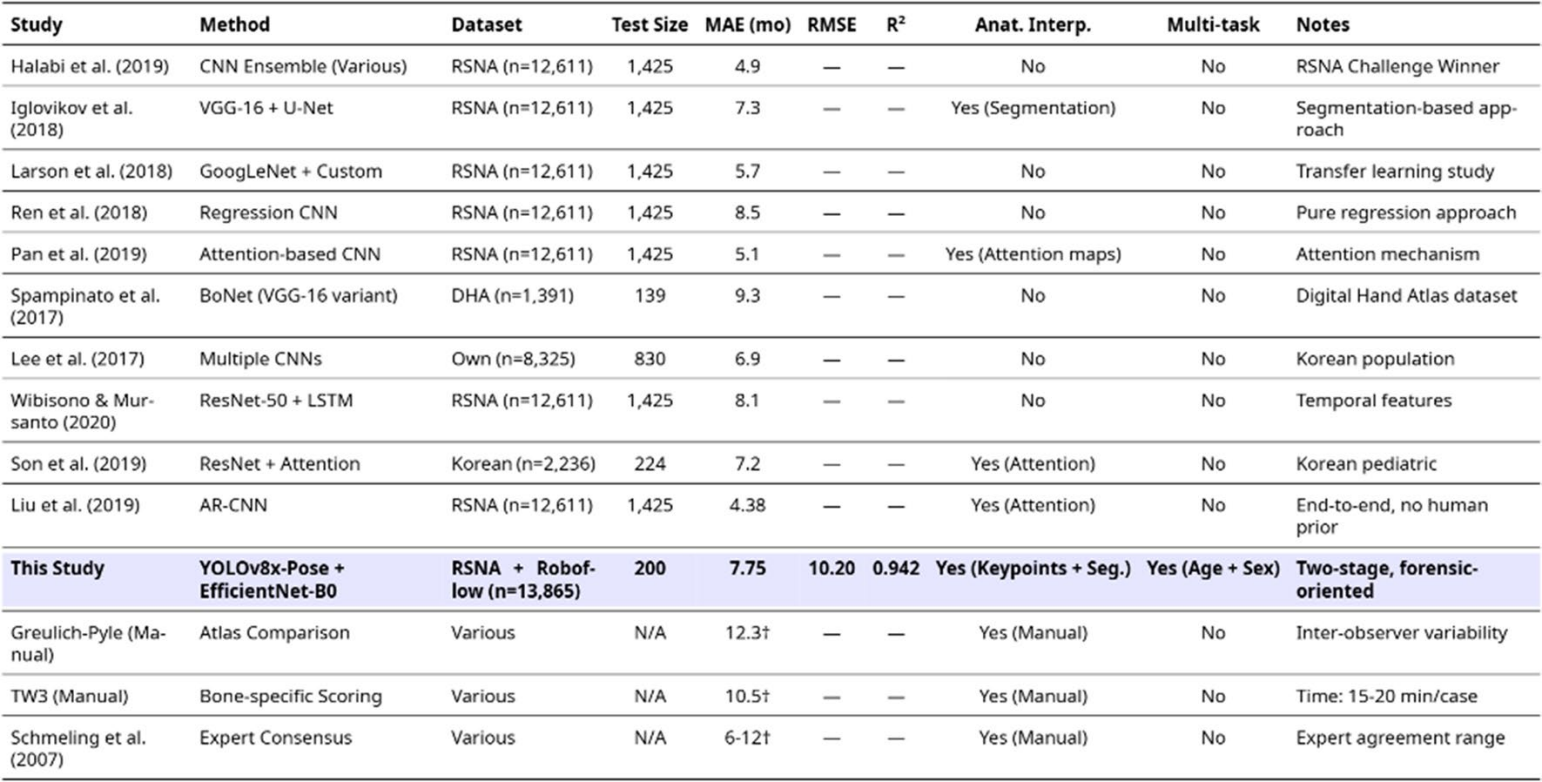
Benchmark comparison with state-of-the-art methods

**Table 9:** Comparative performance of sex estimation methods across skeletal regions

Study	Skeletal Region	Method	Dataset Size	Accuracy (%)	Precision (%)	Recall (%)	F1-Score (%)	Cohen’s κ	Sexual Dimorphism Level	Notes
Bewes et al. (2019)	Skull	CNN	1,200	95	—	—	—	—	High	High dimorphism region
Oner et al. (2019)	Pelvis	SVM + CNN	800	96	—	—	—	—	High	Pelvic CT scans
Garvin et al. (2014)	Pelvis	Morphological	300	90.5	—	—	—	0.81	High	Traditional morphometrics
Grabherr et al. (2018)	Pelvis (CT)	Deep Learning	600	97.2	—	—	—	—	High	Postmortem CT
Klales et al. (2020)	Skull (CT)	CNN	1,500	92	—	—	—	—	High	3D cranial features
Mahfouz et al. (2017)	Various	CNN	2,000	93.5	—	—	—	—	Mixed	Multi-region analysis
**This Study**	**Hand-Wrist**	**EfficientNet-B0**	**13,865**	**94.0**	**94.1**	**94.1**	**94.1**	**0.88**	**Minimal**	**First comprehensive DL study for hand**
Krogman & Iscan (1986)	Hand	Manual Assessment	Various	85	—	—	—	—	Minimal	Traditional method baseline

### Critical comparisons

Our study’s MAE of 7.75 months positions competitively among automated deep learning approaches: slightly higher than Halabi et al.‘s RSNA Challenge-winning ensemble (4.9 months) and Iglovikov et al.‘s segmentation-based method (7.3 months), but superior to Ren et al.‘s regression CNN (8.5 months). Importantly, our system provides anatomical interpretability through pose estimation and segmentation—a feature absent in most competing methods. Compared to manual assessment, our MAE substantially outperforms the Greulich-Pyle method’s inter-observer variability (12.3 months; Schmeling et al., 2007) and falls within the lower range of radiologist agreement (6–12 months; Bull et al., 1999).

For sex classification, our 94% accuracy from hand-wrist radiographs—a region with minimal sexual dimorphism—achieves competitive performance relative to methods utilizing high-dimorphism skeletal regions: Bewes et al. (2019) reported > 95% accuracy from skull analysis. This finding is particularly significant for forensic applications, as hand remains are more frequently recovered intact in fragmentation scenarios compared to skulls or pelves.

### Independence from soft tissue

The YOLOv8x-Seg module, which is part of the system, precisely isolates bone regions, enabling the analysis to focus solely on skeletal features.

Validation Results: Achieving high accuracy (MAE 7.75 months) from predictions made solely on segmented bone regions strongly supports that the model bases its decisions on bone morphology rather than soft tissue shadows.

Implications for Decomposed Remains: In forensic scenarios (e.g., drowning, fire, or prolonged decomposition), soft tissue is typically absent or severely degraded. The findings of this study demonstrate that radiographic skeletal analysis maintains validity in such situations. The automatic segmentation process mimics the examination of isolated skeletal elements in a forensic laboratory. The model’s reliance on bone morphology reveals that the method’s foundation is consistent with traditional forensic anthropological principles.

### Forensic age categories and practical implications

#### Forensic age determination needs

- 0–5 years: Infant and young child distinction (child abuse investigations).
- 5–10 years: Childhood period (criminal responsibility age limits vary globally between 7 and 14 years).
- 10–15 years: Onset of adolescence (unaccompanied asylum-seeking children).
- 15–19 years: Legal adulthood threshold (18 years), military service and human trafficking cases.

#### Assessment of the 18-year threshold

The MAE (8.18 months) obtained in the 15–19 age group indicates an uncertainty interval of approximately ± 8 months around this critical legal threshold. Therefore, rather than the model providing a categorical “adult” or “child” decision, presenting a probabilistic report would be a more accurate approach (e.g., “there is a 90% probability that the individual is older than 18 years”). This approach is also consistent with the guidelines of organizations such as the European Asylum Support Office (EASO) that age estimates should always be reported with an uncertainty interval [36].

#### Study limitations and future research

### Dataset-related limitations

- Population Representation
  - Training Data: The RSNA dataset used in this study primarily represents the North American population, while the Roboflow dataset represents a mixed population of unspecified origin.
  - Forensic Reality: DVI operations typically involve global and highly diverse populations (e.g., 2004 Asian tsunami, Mediterranean migrant tragedies). There is a possibility that the model may show lower performance in populations with different geographic origins and dietary habits due to differences in skeletal maturation rates. The model’s generalization ability needs to be externally validated on forensic datasets collected from different populations.

b) Absence of Pathology and Trauma

#### Exclusion

Training datasets exclude conditions such as fractures, bone dysplasias, or malnutrition.

#### Forensic frequency

The presence of antemortem trauma findings is a common occurrence in forensic cases [37].

Research Need: Testing the model’s performance on trauma-affected hand radiographs such as healed fractures or malunion is an important step for future studies.

c) Taphonomic Effects (Postmortem Changes)

#### Current data

The study uses “fresh” radiographs taken from living individuals.

#### Forensic context

Forensic cases frequently involve bones that have been burned, submerged, or buried and subjected to taphonomic changes.

#### Validation need

The model needs to be tested on more challenging data such as postmortem CT scans obtained from decomposed hands. However, access to such datasets is limited due to ethical and practical challenges.

Potential Advantages Over Traditional Methods:

1. Speed: Analysis time of < 5 s per radiograph is far below the manual Greulich-Pyle method (15–20 min).
2. Scalability: Batch processing capability can increase efficiency in mass disasters with hundreds of victims.
3. Objectivity: Eliminates inter-observer variability (ICC 0.993 vs. 0.70–0.85 for manual methods), which is important for legal admissibility.
4. Remote Analysis: A cloud-based system could enable immediate analysis of radiographs taken in the field by experts in a central laboratory.

## Conclusion

This study presents a two-stage deep learning system for automatic skeletal age and sex estimation from hand radiographs, specifically designed for generating biological profile preliminary reports to facilitate antemortem-postmortem (AM-PM) reconciliation in Disaster Victim Identification (DVI) operations. The system addresses a critical gap in forensic practice: while hand-wrist radiographs are abundantly archived in orthodontic clinics and medical facilities worldwide, no standardized rapid method has existed to generate comparable biological profiles from recovered postmortem hand remains.

### Methodological contribution

The proposed two-stage approach provides three fundamental advantages over single-stage “end-to-end” models: (1) Anatomical interpretability through landmark and segmentation visualization, meeting Daubert criteria for court admissibility; (2) Independence from soft tissue through bone segmentation (Dice > 92%), enabling applicability to decomposed remains; (3) Rapid AM database filtering capability, reducing potential AM-PM matches by approximately 85% before DNA analysis.

### Technical performance

The system, trained with 13,865 radiographs from RSNA and Roboflow datasets, achieves:

- Skeletal Age: MAE 7.75 months (95% CI: 6.82–8.68), R² 0.942, ICC 0.993.
- Sex Classification: 94% accuracy, Cohen’s κ 0.88 (“almost perfect” agreement).
- Processing Speed: <5 s per radiograph.

### Forensic application

The primary utility lies in the DVI Reconciliation Phase, where biological profiles generated from postmortem hand radiographs can be systematically compared against antemortem medical archives. In mass fatality incidents with hundreds of victims, this automated profiling enables rapid stratification of potential matches, significantly reducing the cost and time required for confirmatory DNA analysis.

This automated system provides an objective, rapid, and scalable tool for biological profiling from hand radiographs, particularly valuable when only isolated hand remains are recovered (fragmentation, thermal destruction, aquatic decomposition). The method’s reliance on skeletal morphology alone, validated through segmentation architecture, bridges clinical and forensic contexts and supports deployment in field DVI operations.

## Data Availability

The source code for the two-stage deep learning system is publicly available at https://github.com/ccatak/hand-wrist-age-sex-estimation under MIT License. The datasets analyzed during the current study are available in the RSNA Pediatric Bone Age Challenge repository (https://www.rsna.org/education/ai-resources-and-training/ai-image-challenge/rsna-pediatric-bone-age-challenge-2017) and the Roboflow Universe HandBone Segmentation repository (https://universe.roboflow.com/handbone/handbone-segmentation). Pre-trained model weights are available upon request from the corresponding author.
